# Managing Anesthesia for a Patient with Takayasu Illness

**DOI:** 10.1155/2023/2852203

**Published:** 2023-01-05

**Authors:** Gabriela Freitas, João Luiz Guerra, Cesar Henrique Fornero, Marina Delgado

**Affiliations:** Hospital das Clínicas de Belo Horizonte, Universidade Federal de Minas Gerais, Belo Horizonte, Minas Gerais, Brazil

## Abstract

This article intended to provide an overview of the anesthetic management for a patient with Takayasu arteritis, an uncommon and challenging disease. Despite the limited literature, it becomes more evident that there is no right answer for all patients. Considering that, it is important to take into account the severity and specific features of the underlying disease to decide the best anesthetic approach. In this context, an adequate preoperative assessment focusing on cardiovascular function becomes essential. Adequate cardiovascular monitoring is also essential in the intraoperative period. A multidisciplinary team should be involved in the perioperative period to provide the best care possible and improve patient outcomes. This case illustrates a successful hysterectomy in a patient with Takayasu arteritis, pointing out the pathophysiologic considerations and discussing the means to reduce the perioperative risk. The particularities described in this case report may help other physicians choose the best strategy when facing challenging patients similar to the one described.

## 1. Introduction

Takayasu arteritis is an idiopathic (possibly autoimmune), chronic, inflammatory, and progressive disease that leads to straightening, occlusion, and aneurysms of the systemic and pulmonary arteries [1]. There is granulomatous infiltration of the adventitia and media, followed by a sclerotic phase [2]. The course of this disease fluctuates, as it progresses to exacerbation and remission periods [3]. It affects mainly the aorta and its branches, and the subclavian artery is among the most common vessels affected [4]. The blood flow in the affected areas is reduced, and because of that, the patient may not have palpable pulses. This disease is also known as “pulseless women disease” [2]. The prevalence is higher among young women, between 10 and 40 years of age, and the majority of the cases are found in Asia [3]. Internationally, it affects 6 people per 1000 [2]. The diagnosis is based on clinical findings, magnetic resonance angiography, and computed tomography angiography [3]. There are no specific laboratorial tests available for diagnosis, but disease activity can be monitored with the erythrocyte sedimentation rate [2]. This arteritis can be classified into four types according to the vessels affected: type 1 involves the aortic arch and its branches, type 2 affects the thoracic and abdominal aorta, type 3 is a mixture between types 1 and 2, and type 4 involves the pulmonary artery [5]. The treatment is based on glucocorticoids and other adjuvants that include methotrexate, azathioprine, mycophenolate, and leflunomide [3]. Patients with arteritis may undergo surgical procedures to treat aneurysms and arterial occlusions [3]. Revascularization procedures should be avoided during the active phase of the disease to reduce the risk of restenosis of the vessels [3]. The most common causes of death include heart failure, myocardial infarction, and stroke [5].

## 2. Case Report

A 43-year-old woman with uterine leiomyomas and severe anemia due to bleeding was admitted to the hospital for anemia control and subsequent hysterectomy. This patient has Takayasu arteritis, as well as bilateral neurosensorial deafness, arterial hypertension, and dyslipidemia. This patient was taking enalapril 5 mg once a day, amlodipine 10 mg once a day, hydrochlorothiazide 25 mg once a day, acetylsalicylic acid 100 mg once a day, folic acid 5 mg twice a week, prednisone 5 mg once a day, methotrexate 25 mg once a week, and adalimumab 40 mg every fourteen days. Previous exams revealed urea 15 mg/dL, creatinine 0.75 mg/dL, normal glomerular filtration rate, hemoglobin of 11.9 g/dL, platelets of 493 × 10^9^ (normal range = 150−500 × 10^9^), activated partial thromboplastin time (aPPT) at 25.2 (normal range = 25.1−36.5 s), a prothrombin time at 10.6 (normal range = 9.4−12.5 s), and a fibrinogen of 486 mg/dl. Thyroid hormones were within normal limit which are TSH of 0.72 *µ*UI/ml and free T4 of 1.0 ng/dL. Cardiac catheterization and an ergometric test showed no signs of cardiac ischemia. No pulmonary hypertension, echocardiography, or respiratory status alterations were present. In the preoperative period, this patient was classified as a Lee class 1 risk patient for cardiac events, according to the Lee score. This patient had a probable Takayasu type 1 disease in the remission period, which presented itself with a severe occlusion in the proximal part of the left subclavian artery and there was also stenosis of the common and internal left carotid and left iliac arteries. The rheumatology team suggested that adalimumab should be suspended for 30 days before surgery. Prednisone and methotrexate should be continued. On the day of the procedure, the patient was admitted stable and oriented and monitored with electrocardiography, pulse oximetry, and noninvasive pressure in the right arm (arterial pressure was not mensurable in the left arm). An invasive blood pressure monitor was not used because of the greater risk of complications associated with this procedure in this scenario. The spinal anesthesia was performed with a 27G Quincke needle, at L4-L5, reaching a T4 level of blockade. A bolus dose of 17.5 mg of hyperbaric bupivacaine and 80 *µ*g of morphine with a total volume of 3.9 mL were injected in the neuraxial blockade. Sedation was performed with midazolam 5 mg, fentanyl 50 *µ*g, and ketamine 20 mg. Ramsay sedation scale level 2 was obtained. This allowed the anesthesiologist to clinically monitor neurological status. A bolus dose of 75 mg of hydrocortisone was administered during the anesthetic procedure. A total of 1000 mL of ringer lactate was administered (half of this quantity was administered before spinal anesthesia). The procedure occurred with no complications, and the patient recovered well. She was discharged home after 2 days.

## 3. Discussion

Takayasu arteritis is a pan-endarteritis [2] that can generate symptoms, such as uncontrolled hypertension (multiple causes), fever, fatigue, loss of weight, neuropathy, anemia, hypoalbuminemia, and nephropathy [1, 6]. Various other symptoms can also be present at the time of the anesthetic procedure, such as visual disturbances, seizures, and neurological deficits caused by previous cerebral vascular injury [6]. In the cardiovascular system, it is important to evaluate the presence of valvular dysfunction, arrhythmia, ischemic heart disease, and aortic dissection [1, 6, 7]. In the respiratory system, the patient may present with pulmonary hypertension and ventilation-perfusion defects [6]. Other diseases can also be present, such as ankylosing spondylitis and rheumatoid arthritis [2]. It is essential to consider all those possible clinical manifestations to proceed with a safe anesthetic management and adequate preanesthetic prepare. A proper preoperative assessment should be performed, including the evaluation of thyroid function before the procedure, since there may be an associated autoimmune disease affecting the thyroid gland [2]. In the intraoperative period, it should be considered the use of a stress corticosteroid dose [1], according to the chronic dose that the patient is using and to the risk of the procedure. In addition, the correct positioning of the head is essential because hyperextension can cause an important reduction in the carotid blood flow [8]. End tidal carbon dioxide pressure should be kept in a normal range to avoid cerebral vasoconstriction and ischemia if general anesthesia is performed [8]. Regional anesthesia has been used successfully, although there are some concerns regarding the use of anticoagulants and the possible impact of hypotension in these patients [1, 6]. This anesthetic technique is relatively contraindicated if spinal cord hypoperfusion [2] is present, and it should also be avoided if there are important differences between the arterial pressure of the upper and lower limbs, since it can limit regional blood flow in an unpredictable way [9]. It is also important to administer fluids before regional anesthesia, avoiding hypovolemia and significant hemodynamic fluctuations [9, 10]. Independently of the anesthetic technique and of the drugs used, priority should be to keep blood pressure in an adequate level. Both hypotension and hypertension should be avoided [1, 5]. Regarding the arterial pressure monitoring, it can be hard to use noninvasive pressure methods because there may be no pulse in the upper limbs. If there is a difference greater than 20 mmHg between one arm and the other, both of them should be monitored [1, 10]. Complications of invasive arterial pressure monitoring are more common in these patients and require the anesthesiologist to be always attentive [1]. Blood pressure measurements in the extremities may not be accurate and differ from aortic and cerebral perfusion pressures. In conclusion, a patient with Takayasu arteritis can be very difficult to manage, and it is important that the anesthesiologist be aware of its particular features to proceed with a secure anesthesia.
